# Heat Shock Proteins in Lymphoma Immunotherapy

**DOI:** 10.3389/fimmu.2021.660085

**Published:** 2021-03-18

**Authors:** Zarema Albakova, Yana Mangasarova, Alexander Sapozhnikov

**Affiliations:** ^1^Department of Biology, Lomonosov Moscow State University, Moscow, Russia; ^2^Department of Immunology, Shemyakin and Ovchinnikov Institute of Bioorganic Chemistry RAS, Moscow, Russia; ^3^National Research Center for Hematology, Moscow, Russia

**Keywords:** heat shock proteins, lymphoma, CAR T, CAR NK, checkpoint inhibitors

## Abstract

Immunotherapy harnessing the host immune system for tumor destruction revolutionized oncology research and advanced treatment strategies for lymphoma patients. Lymphoma is a heterogeneous group of cancer, where the central roles in pathogenesis play immune evasion and dysregulation of multiple signaling pathways. Immunotherapy-based approaches such as engineered T cells (CAR T), immune checkpoint modulators and NK cell-based therapies are now in the frontline of lymphoma research. Even though emerging immunotherapies showed promising results in treating lymphoma patients, low efficacy and on-target/off-tumor toxicity are of a major concern. To address that issue it is suggested to look into the emerging role of heat shock proteins. Heat shock proteins (HSPs) showed to be highly expressed in lymphoma cells. HSPs are known for their abilities to modulate immune responses and inhibit apoptosis, which made their successful entry into cancer clinical trials. Here, we explore the role of HSPs in Hodgkin and Non-Hodgkin lymphoma and their involvement in CAR T therapy, checkpoint blockade and NK cell- based therapies. Understanding the role of HSPs in lymphoma pathogenesis and the ways how HSPs may enhance anti-tumor responses, may help in the development of more effective, specific and safe immunotherapy.

## Introduction

Lymphoma is a heterogeneous cancer divided into two major types such as Hodgkin lymphoma (HL) and Non-Hodgkin lymphoma (NHL) (1, 2). In oncology research, management of lymphoma stands out as the choice of treatment is largely based on the results obtained from prospective clinical trials (1). Standard treatment regimen includes chemotherapy and radiation therapy for the treatment of HL and chemotherapy combined with anti-CD20 antibodies for NHL patients, reaching the cure rate of 80-90% (1, 2). Even though the response rate is high, treatment-related toxicity such as induction of second malignancy and cardiotoxicity is of a major concern (1). Following initial treatment, 10-30% of lymphoma patients develop refractory or recurrent (r/r) disease which is treated with high-dose chemotherapy followed by an autologous hematopoietic stem cell transplantation (ASCT) (1, 2). The overall goal of current and emerging treatments for HL and NHL is to cure disease and minimize treatment-related toxicity (1, 2). Current treatments for lymphoma patients are summarized in **Table 1**. Recently approved treatments for r/r HL and NHL subtypes include anti-CD30 antibody-drug conjugate brentuximab vedotin, PD1 inhibitors (pembrolizumab and nivolumab), Bruton’s tyrosine kinase inhibitors (ibrutinib and acalabrutinib), phosphoinositide 3-kinase γ and/or δ inhibitors (idelalisib, copanlisib and duvelisib) and CD19 chimeric antigen receptor (CAR) T cell therapy (tisagenlecleucel and axicabtagene ciloleucel) (**Table 1**) (1, 2, 29).

**Table 1:** Current treatments for HL and NHL.

Hodgkin lymphoma		
Lymphoma type	Standard treatment regimen	Refs
cHL	Chemotherapy + ISRT	(1, 3, 4)
r/r cHL	High-dose chemotherapy +ASCT	(1, 4–7)
NLPHL	Rituximab	(3, 8)
	**New agents**	
cHL, including r/r cHL	Brentuximab vedotin	(1, 3, 9)
	Nivolumab	(3, 10)
	Pembrolizumab	(3, 11)
**Non-Hodgkin lymphoma**		
	**Standard treatment regimen**	
NHL, including r/r NHL	Rituximab+chemotherapy	(2)
	Lenalidomide+Rituximab	(2)
	High-dose chemotherapy +ASCT	(2)
	**New agents**	
PTCL	Brentuximab vedotin	(12)
CLL/SLL	Ibrutinib+rituximab	(13, 14)
CLL/SLL; MCL	Acalabrutinib	(15–20)
FL and SLL	Idelalisib	(21)
FL	Copanlisib	(22)
r/r CLL/SLL	Duvelisib	(23)
r/r primary mediastinal BCL	Pembrolizumab	(24, 25)
r/r DLBCL	Tisagenlecleucel	(26)
r/r DLBCL	Axicabtagene ciloucel	(27, 28)

ISRT, Involved site radiation therapy; ASCT, autologous haemotopoietic stem cell transplantation; cHL, classic Hodgkin lymphoma; NLPHL, nodular lymphocyte-predominant Hodgkin lymphoma;r/r, Refractory or recurrent disease; PTCL, peripheral T-cell lymphoma; CLL, chronic lymphocytic leukemia (CLL); SLL, small lymphocytic lymphoma; MCL, mantle cell lymphoma.

Heat shock proteins (HSPs) are molecular chaperones highly expressed in various types of cancer. HSPs are classified into several families such as HSP110, HSP90, HSP70, HSP40, chaperonins and HSPB (30). HSPs are largely known for their role in blocking apoptosis which was further translated into development of HSP inhibitors (31–49). Along this line, Kamal and colleagues showed that HSP90 inhibitor 17-allylamino-17-demethoxy-geldanamycin (17-AAG) selectively targets cancer cells (50). In light of the reported, several HSP90 inhibitors such as alvespimycin (NCT01126502), luminespib (NCT01485536), PU-H71 (NCT01581541) and SNX-5422 (NCT02914327) currently are assessed in clinical trials for the treatment of lymphoma patients. Furthermore, HSPs showed to be potent immune system activators through the induction of cytotoxic CD8+T cell response (51–56). Several HSP-based vaccines have been evaluated in clinical trials (57–61). Specifically, the efficacy and safety of HSPPC-96 vaccine, which is an autologous gp96 heat shock protein-peptide complex vaccine, was assessed in patients with indolent non-Hodgkin lymphoma (62). Inspired by the ability of HSPs to induce immune responses, Li and colleagues developed a novel nanovaccine that mimics HSPs, so it can be used to stimulate anti-tumor immune responses (55). Additionally, numerous studies have assessed extracellular HSPs derived from various liquid biopsies (serum, plasma, urine, plasma/serum/urine-derived exosomes) as potential cancer biomarkers [reviewed in (31)] (63–71).

Novel emerging immunotherapy approaches involve CAR T cell therapy, checkpoint inhibitors and NK cell-based therapies that are aimed at improving effectiveness in treating of lymphoma patients. In this review, we focus on the role of HSP family in Hodgkin and Non-Hodgkin lymphoma. Since the mechanism of apoptosis and immune modulation are the key features in lymphoma pathogenesis, it is of particular interest to explore the contribution of HSPs in this process. We explain how the understanding of the cross-talk between tumor microenvironment (TME) and malignant cells and the role of HSPs in this process may help to improve emerging treatments for lymphoma patients.

## HSPs in Hodgkin Lymphoma

Hodgkin lymphoma (HL) is a B cell lymphoma divided into classic HL (cHL) which accounts for the majority of the cases and nodular lymphocyte-predominant HL (NLPHL) (1). Histologically, cHL is classified into four types such as nodular sclerosis HL (NSHL), mixed cellularity HL (MCHL), lymphocyte-rich HL (LRHL) and lymphocyte-depleted HL (LDHL) (1).

**Figure 1:**
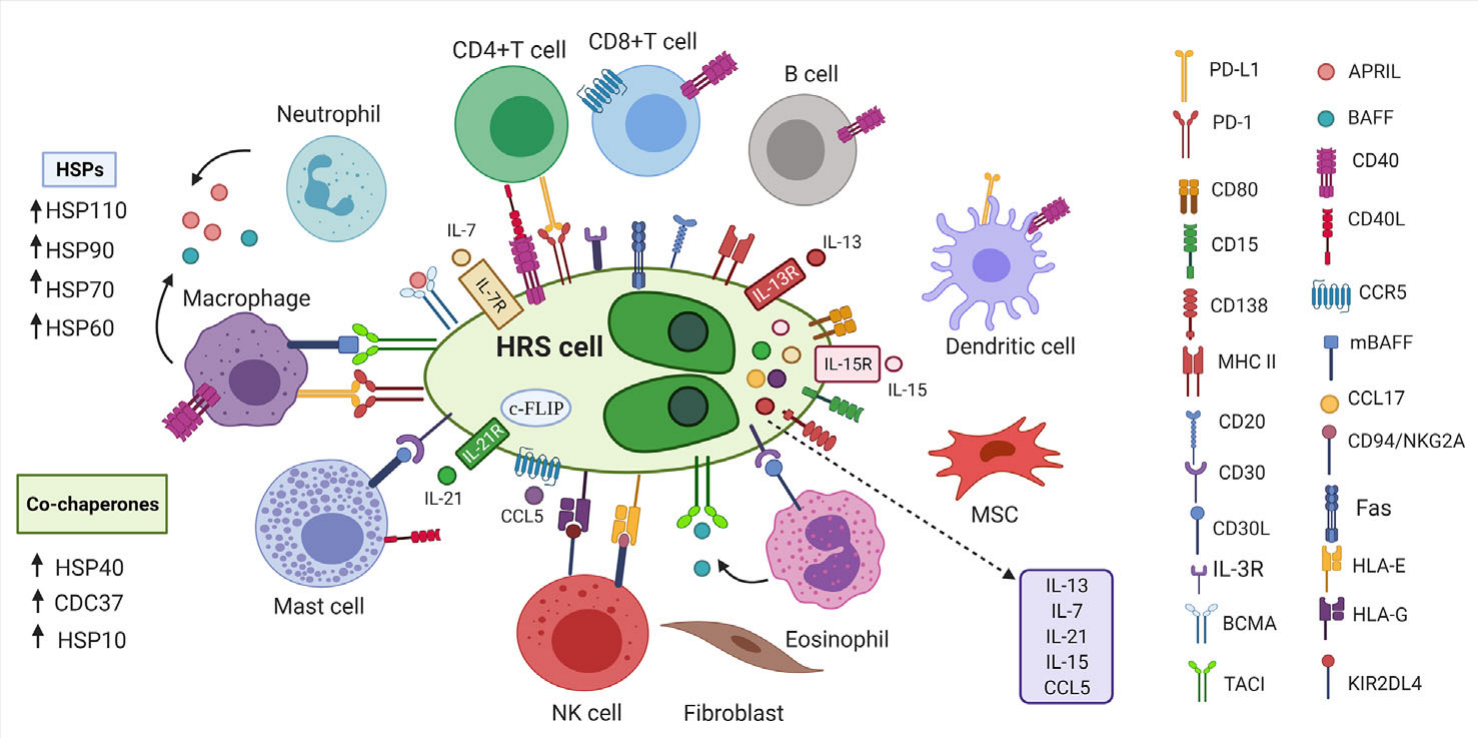
Tumor microenvironment in cHL. HRS cells are surrounded by non-malignant immune and stromal cells. Inflammatory cells secrete cytokines, tumor necrosis family members (CD40L, CD30L) and other molecules (APRIL, BAFF) that bind to the proteins on the surface of HRS cells to promote growth and survival of HRS cells (1, 73). HRS cells express various markers of B cells, T cells, myeloid markers, markers of dendritic cells (74). HRS cells express PD-L1 to escape anti-tumor responses (75). HRS cells express Fas, but avoid FasL-mediated apoptosis by overexpressing c-FLIP (75–77). HRS cells express FasL leading to apoptosis of Fas-expressing NK cells (76, 78). HSP chaperones and their corresponding co-chaperones are highly expressed in HRS cells, which further contribute to immunosuppressive TME (79, 80). HRS, Hodgkin and Reed-Sternberg cells; BCMA, B cell maturation antigen; APRIL, a proliferation-inducing ligand; BAFF, B-cell activating factor;PD-L1, programmed death ligand 1; PD-1, programmed death 1; CD30L, CD30 ligand; CD40L, CD40 ligand; CCL5, CC-chemokine ligand 5; IL-3R, interleukin-3 receptor; TACI, transmembrane activator and calcium-modulator and cyclophilin ligand interactor; HSP, Heat shock protein; MSC, mesenchymal stromal cells; mBAFF, membrane-bound B-cell activating factor; MHC II, Major histocompatibility complex class II; HLA-E/G, Human leukocyte antigen- E/G; KIR2DL4, killer cell immunoglobulin-like receptor family member; c-FLIP, cellular FLICE-inhibitory protein; cHL, classic Hodgkin lymphoma; NK cells, natural killer cells.

cHL is characterized by the presence of malignant Hodgkin and Reed-Sternberg (HRS) cells that constitute minor population (~1%) of the tumor mass (72). The majority of infiltrate surrounding HRS cells is represented by different types of non-malignant immune cells such as dendritic cells, macrophages, lymphocytes, mast cells, neutrophils, eosinophils and fibroblasts which form tumor microenvironment (TME) (1, 72). Even though, HRS cells are germinal center (GC)- derived B cells, they resemble immunophenotype that does not associate with any known cells of hematopoietic origin (**Figure 1**) (74). Specifically, HRS cells rarely express typical B-cell lineage markers such as CD19, CD20, CD22, CD79, CD79B and instead express markers of dendritic cells (CD83), myeloid markers (CD15) and T cell markers (CD2, CD3, CD4) (1, 74, 75, 81). Additionally, members of tumor necrosis factor family namely CD30 and CD40 are expressed on HRS cells (75). It is interesting to point out that cHL TME is composed of variable cellularity that is different in each cHL subtype (1). As an example, NSHL is rich in fibroblast-like cells and fibrosis, MCHL is composed of B cells, T cells, neutrophils, histiocytes, plasma cells and mast cells, LRHL is characterized by HRS cells surrounded by mantle zone B cells and histiocytes whereas LDHL predominantly consists of CD4^+^T cells, histiocytes and fibrosis (1).

Nuclear factor- kappa B (NF-kB) is constitutively activated in HRS cells (1, 82). Engagement of CD40, CD30, RANK and BCMA/TACI with their cognate ligands showed to activate NF-kB leading to increased production of IL-6, IL-8, CCL5, IFNγ and IL-13 (75). During activation process, the IKK complex, which is composed of two kinase subunits IKKα, IKKβ and a regulatory subunit IKKγ, phosphorylates IkBα, an inhibitor of NF-kB, allowing NF-kB translocation to the nucleus and activation of genes responsible for B-cell proliferation and survival (73). Notably, treatment with HSP90 inhibitor geldanamycin resulted in inactivation of NF-kB and IKK activity in HRS cell lines (83). HSP90 showed to stabilize subunits of IKK complex (IKKα and IKKβ) and protect them from proteasomal degradation (83).

Another important signaling cascade which is constitutively activated in HRS cells is Janus kinase (JAK)-signal transducer and activator of transcription (STAT) (1). Activation of JAK-STAT pathway leads to hyperphosphorylation of STAT proteins (1). In particular, STAT3, STAT6 and STAT5 showed to be constitutively phosphorylated in cHL cell lines (84–86). Intriguingly, Schoof and coworkers reported that pharmacological blocking of HSP90 inhibited the phosphorylation of STAT1, STAT3, STAT5 and STAT6 in cHL cell lines (84). Overall, HSP90-targeting agents may be a promising strategies in cHL where deregulated NF-kB and JAK-STAT signaling pathways play a major role in cHL pathogenesis.

Phosphatidylinositol 3-kinase (PI3K)-serine/threonine protein kinase (AKT) pathway is also constitutively activated in HRS cells as a result of activation of multiple receptor tyrosine kinases (RTKs) (1, 87). RTKs such as platelet-derived growth factor receptor A (PDGFRA), discoidin domain-containing receptor 2(DDR2),tyrosine kinase receptor A (TRKA) and TRKB showed to be aberrantly expressed in HRS cells of HL patients, while no expression of these RTKs was observed in normal B cells or B-cell NHL cells (87). Furthermore, aberrant expression of mitogen-activated protein kinase (MAPK)/ERK has been reported in HL (88).In light of the reported, HSP90 inhibitor 17-AAG showed to deplete AKT and inhibit extracellular signal-regulated kinase (ERK) phosphorylation, leading to growth arrest and apoptosis in HL cell lines (89) (88). This was further supported by the finding that HSP90 inhibitor celastrol induced anti-tumor effects in HRS cells by downregulating RAS, ERK1/2 and c-Fos (90). In another experiment, inhibition of HSP90 by geldanamycin induced apoptosis in HRS cells with wild-type *IkBα* in p53-independent manner (91). Taken together, these observations suggest that targeting AKT, NF-kB and MAPK/ERK pathways with HSP90 inhibitors may prove effective in HL treatment.

In addition to unique immunophenotype and multiple deregulated signaling pathways, HRS cells express high level of HSPs. Hsu and colleagues assessed HSP expression of formalin-fixed, paraffin-embedded tissues derived from patients with different cHL subtypes (80). High cytoplasmic expression of HSP90 and HSP60 in HRS cells was found in NSHL, MCHL, LRHL and LDHL (80). By contrast, no cytosolic HSP27 expression was found in HRS cells in LRHL and low expression in LDLH while 20% of patients with NSHL and MCHL showed strong HSP27 expression (80). Later, Santon and co-workers used tissue microarray to analyze immunohistochemical expression of HSPs in HRS cells of cHL patients (79). More than 90 percent of cHL patients in HRS cells showed high cytoplasmic expression of HSP60, HSP10, HSP90, and CDC37, nuclear HSF1 whereas HSP110 showed to be highly expressed in nucleus and cytoplasm of HRS cells (79). Positive cytoplasmic staining of HSP70 and cytoplasmic/nuclear expression of HSP40 was observed in 78% of cHL patients whereas 54% had positive cytoplasmic expression of HSP27 (79). Expression of HSP90 and HSP70 positively correlated with expression of their co-chaperones CDC37 and HSP40, respectively (79). Furthermore, expression of HSP40 positively correlated with p53, caspase 9 and cellular FLICE-inhibitory protein (c-FLIP) whereas HSP70 expression correlated with caspase 3 (79). In another study, high cytoplasmic expression of HSP60 was observed in HRS cells in 100% of NSHL and MCHL cases (92).

## HSPs in Non-Hodgkin Lymphoma

Non-Hodgkin lymphoma (NHL) is comprised of B-cell lymphoma, accounting for the majority of the NHL lymphoma subtypes, while other NHLs include T-cell lymphoma and NK-cell lymphoma (93). NHL is classified into indolent (slow-growing) and aggressive (fast-growing) lymphoma. The most common indolent lymphoma is follicular lymphoma (FL), while other slow-growing lymphoma subtypes include marginal zone lymphoma (MZL), chronic lymphocytic leukemia (CLL)/small lymphocytic lymphoma (SLL) and lymphoplasmacytic lymphoma (94). The most common aggressive NHL subtype is represented by diffuse large B-cell lymphoma (DLBCL), while other aggressive lymphoma subtypes include mantle cell lymphoma (MCL), Burkitt lymphoma (BL) and primary effusion lymphoma (94).

Lymphoma cells largely depend on microenvironment for their growth and survival (95). Continuous signaling from B cell receptor (BCR), immune and stromal cells are required to support proliferation activity and survival of lymphoma cells. BCR is required for B cell survival and the loss of BCR results in B cell death (95, 96). BCR activation by self-antigens showed to be a driving force in various NHL subtypes (97–99). Moreover, some subtypes of DLBCL carry genetic mutations that activate BCR signaling, including mutation in *CD79B* and *CARD11*, where the latter mutation leads to constitutive activation of NF-kB in activated B cell-like (ABC) subtype of DLBCL (95, 97, 100–102). Notably, Walter and colleagues demonstrated that HSP90 and its client protein spleen tyrosine kinase (SYK) are required for tonic BCR signaling in BL lymphoma, suggesting potential use of HSP90 as potential target for BL lymphoma treatment (103, 104). Additionally, HSP90 showed to stabilize BCR kinases such as Bruton tyrosine kinase (BTK), SYK, LYN and AKT in chronic lymphocytic leukemia cells (105). Recent studies have added more insight into the role of HSP90 in BCR signaling in NHL subtypes. Jacobson and colleagues reported that HSP90 inhibition led to the complete loss of BTK and IKKα and downstream loss of phosphorylated ERK1/2 in mantle cell lymphoma cell lines (106).Moreover, HSP90 inhibitor showed to downregulate BTK in cells expressing BTK C481S mutation, which was found to be associated with resistance to BTK inhibitor ibrutinib in MCL and CLL patients (106–108). Importantly, Cerchietti and colleagues showed that HSP90 interacts with B-cell lymphoma-6 (Bcl-6) which was further supported by the finding that HSP90 inhibitor PU-H71 selectively killed Bcl-6-dependent DLBCL cells (109). Subsequently, Goldstein and co-workers used PU-H71 and tumor-enriched HSP90 (teHSP90) complexes derived from DLBCL cell lines to show that LYN, SYK, BTK and phospholipase C γ 2(PLCγ2) are dependent on teHSP90 (110). Furthermore, treatment with PU-H71 showed to disrupt BCR signaling, calcium influx and NF-kB activity, resulting in cell growth inhibition (110). Additionally, PU-H71 in combination with ibrutinib led to the killing of lymphoma cells, suggesting that combinatorial therapeutic approach may be more effective in NHL patients (110).

In addition to continuous BCR signaling, lymphoma cells require additional signals to survive. Early experiments in establishing NHL cell lines showed that FL cells require signals from T cells for CD40-mediated interaction and IL-4 stimulation for sustained proliferation of lymphoma cells (95, 111–113). Furthermore, in NHL subtypes myeloid cells secrete high level of B cell- activating factor (BAFF) and a proliferation inducing ligand (APRIL) that are critical for survival and differentiation of B cells (73, 95, 114–117). In addition to the signals provided by immune cells, the cross-talk between stromal cells and FL cells plays important role for the growth of FL B cells [reviewed in (118)].

Members of HSP family showed to be highly expressed in NHL subtypes. Valbuena and colleagues reported moderate-to-strong cytoplasmic expression of HSP90 in 100% of cases of BL,61% of FL patients, 59% of DLBCL, 38% of nodal MZL and 33% of cases with SLL/CLL and 30% of lymphoplasmacytic lymphoma (119). Weak cytoplasmic expression of HSP90 was observed in 43% of cases with extranodal marginal zone B-cell lymphoma of mucosa-associated lymphoid tissue (119). Patients with T-cell lymphoma showed moderate/strong cytoplasmic expression of HSP90 (119). HSP60 also showed to be highly expressed in DLBCL and high-grade FL whereas no HSP60 was detected in low-grade FL (92). NK/T-cell lymphomas showed positive cytoplasmic expression of HSP60 (92).

Recent studies have emphasized the role of HSP110 in aggressive subtypes of B-cell NHLs such as DLBCL and BL (120). Zappasodi and colleagues demonstrated that inactivation of HSP105/HSPH1 leads to downregulation of c-Myc and Bcl-6 (120).Mechanistically, HSP105 showed to interact with c-Myc and Bcl-6 in nucleus in primary human DLBCL and BL cells, suggesting that HSP105 may function as a chaperone for both c-Myc and Bcl-6 (120).Additionally, higher expression of HSP105 was found in DLBCL expressing c-Myc compared to c-Myc low/negative counterparts (120). In light of the reported, Boudesco and co-workers showed that overexpression of HSP110 resulted in upregulation of NF-kB, whereas silencing of HSP110 donwregulated NF-kB, suggesting that there is an interplay between HSP110 and NF-kB (121). Mechanistically, HSP110 showed to stabilize myeloid differentiation factor 88 (MyD88), leading to chronic NF-kB activation in ABC-DLBCL (121). Therefore, targeting HSP110 may be a promising strategy for the treatment of B-cell NHLs.

Phase II clinical trial was conducted to assess the safety and efficacy of HSP90 inhibitor AUY922 in patients with r/r DLBCL and peripheral T-cell lymphoma (PTCL) (92). Overall, 14 patients with DLBCL and 6 with PTCL were enrolled, 1 patient with DLBCL reached complete response (CR) and 1 patient with PTCL achieved partial response (122). Treatment-related adverse effects included fatigue, visual disturbance that was fully reversible and anemia (122). Authors concluded that HSP90 inhibitors may be a good target in some cases, though, combination with chemotherapeutic agents and histone deacetylase (HDAC) inhibitors may be used to improve anti-tumor activity (122). Several studies assessing combination of HSP90 inhibitors with chemotherapeutic drugs such as fludarabine, doxorubicin, cytarabine, melphalan, or HDAC inhibitors demonstrated promising results in hematological malignancies (122–126). Taken together, NHL subtypes have high expression of specific HSP members, however, use of combinatorial approach in NHL patients warrants further investigation.

## HSPs and Emerging Lymphoma Immunotherapy

### HSPs and CAR T

CAR T therapy involves *ex vivo* expansion and genetic modification of an autologous (self) or allogeneic (donor) T cells that specifically identify and eliminate cognate target ligand (127, 128). CAR consists of antigen-recognition domain represented by a single-chain variable fragment (scFv), hinge, transmembrane and intracellular signaling domains (127). Majority of CARs contain CD3ζ which is critical for T cell receptor (TCR) signaling (129). Due to low CAR T cell activity and persistence, second generation CARs have been developed that integrated co-stimulatory domains derived from CD28 or 4-1BB into the CAR design (128, 129). Importantly, CAR T cells that contain CD28 domains differentiate into effector memory T cells whereas 4-1BB-domain CAR T cells differentiate into central memory T cells (128, 130).

CAR T showed to be effective in the treatment of B-cell malignancies (129). In 2017 the first CAR T immunotherapy tisagenlecleucel (CD19-specific 4-1BB-CAR) was approved by FDA for the treatment of r/r B-cell acute lymphoblastic leukemia (B-ALL) (128). Later, in 2018, axicabtagene ciloucel (CD19-specific CD28-CAR) was approved for the treatment of r/r DLBCL (93). Nevertheless, the main challenge in CAR T therapy now is to find an antigen universally expressed on tumor cells that can be targeted by CAR T. Since HRS cells almost exclusively express CD30, CD30 was proposed as an attractive target for the CAR T therapy. Up till now only 3 studies assessed the efficacy and safety of CD30 CAR T immunotherapy for the treatment of Hodgkin lymphoma (129). Recently, Ramos and colleagues have conducted two phase I/II clinical trials where autologous CD30 CAR Ts were administered to patients with r/r Hodgkin lymphoma after lymphodepletion with fludarabine in combination with either bendamustine or cyclophosphamide (131). The overall response rate (ORR) for 32 patients was 72%, 19 (59%) of which achieved a complete remission (131). It is encouraging that no neurotoxicity was observed. Cytokine release syndrome (CRS) and skin rash that occurred in 10 and 20 patients, respectively, were found to be associated with cyclophosphamide rather than with bendamustine and both spontaneously resolved (131). In another study, Wang and colleagues designed anti-CD30 CAR and conducted a pilot study in patients with r/r Hodgkin lymphoma (132). After lymphodepletion, patients were infused with anti-CD30 CAR Ts. A total of 9 patients received CD30 CAR T infusion, 3 achieved CR, six experienced CRS from which 4 were low-grade and no neurotoxicity was observed (132). Authors also demonstrated promising results in combination therapy where CD30 CAR T treatment was combined with anti-PD-1 antibody (132). Notably, Watanabe and colleagues showed that CD30 induces expression of HSP90α and HSP90β in cHL by activating heat shock factor 1 (HSF1) (133). Since CD30 and HSP90 are overexpressed in cHL, future studies should explore the effect of anti-CD30 CAR T therapy on HSP90 (133).

**Figure 2:**
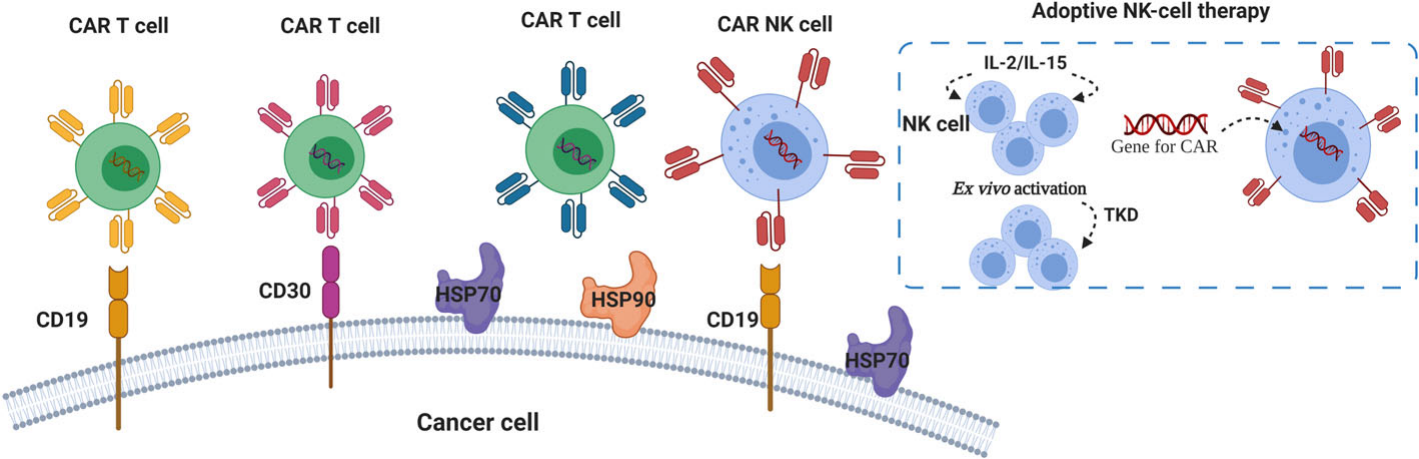
HSPs and CAR T/NK cells in lymphoma immunotherapy. Chimeric Antigens Receptor (CAR) targeting CD19 and CD30 showed promising results in patients with r/r lymphoma (93, 129, 131, 132). Autologous NK cells pre-activated with cytokines (IL-2, IL-15) and 14-mer HSP70-derived peptide (TKD) can be used for *ex vivo* activation of NK cells for the adoptive transfer therapy (137–140). Anti- HSP70 and HSP90 CARs were proposed for specific targeting of membrane-bound forms of HSP70 and HSP90 (134, 136).

The use of CARs against HSP70 was proposed by Smith and colleagues (134). Their invention particularly aims to target membrane-bound form of HSP70, so that specifically HSP70-surface positive tumor cells can be killed (134). Similar to the Smith group, Claffey and co-workers identified heavy chain antibody (HCAb2) that selectively targets HSP90 on malignant cells (135). Based on their findings, authors described an antibody that specifically binds to the cell surface HSP90β isoform (135, 136). Therefore, targeting surface expression of HSPs can be a new promising strategy for the development of more efficient CAR T therapy (**Figure 2**).

Due to on-target/off-tumor toxicity that associated with the use of CAR T therapy, several investigators proposed that the activity of CAR T cells can be controlled by thermal regulation with the use of HSP-based promoters (pHSP) (141, 142). Shapiro and colleagues used genetically engineered circuits pHSP-CAR to induce CAR expression in T cells in response to thermal stimuli (142). Studies assessing the use of pHSP for thermal control of CAR T highlighted that, despite their names, HSP members respond to various cellular stresses such as heat, hypoxia, radiation, heavy metal toxicity and cytokines (142–144). Therefore, the choice of HSP-based promoter should largely depend on the context in which these promoters will be used (142).

Several investigators demonstrated that HSP90 is crucial for the functional activity of T lymphocytes. For example, Bae and coworkers demonstrated that HSP90 inhibition downregulates the expression of CD3, CD4, CD8 as well as CD28, CD40L and αβ receptors and cripples T cell proliferation and interferon-γ (IFN-γ) secretion (145). In another study, pharmacological blocking of HSP90 resulted in decreased expression of Linker for activation of T cells (LAT) in activated T cells (146). Therefore, taking into account that HSP90 is important for the T cell function and phenotype, and that CAR T-containing CD28 and CD3 domains may affect intracellular and extracellular HSP90 expression, further studies should address the effect of CAR T on HSP90 expression.

### HSPs and NK Cell-Based Immunotherapy

The immunosuppressive TME specifically inhibits functional activity and proliferation of NK cells (76). Several investigators reported deficiency in the number of NK cells in the biopsies of cHL patients (76, 147). Moreover, lower number of circulating NK cells were detected in peripheral blood of cHL patients (76, 148, 149). The main goal of NK-based immunotherapy in HL is to reactivate NK cells (150). Boll and colleagues demonstrated that HSP90 inhibitor called BIIB021 combined with doxorubicin and gemcitabine selectively killed Hodgkin lymphoma cells by inhibiting NF-kB activation (150). Moreover, HSP90 inhibition resulted in upregulation of NKG2D ligands such as MHC class I chain-related A (MICA), MICB and ULBP2 on HL cells, making HL cells susceptible to NK-mediated killing (150). In line with that, Fionda and colleagues demonstrated that HSP90 inhibition upregulates MICA and MICB and leads to increased NK cell degranulation in myeloma cell lines (151).

Contrary to T cells, NK cells do not recognize an antigen in the form of MHC-peptide complex, but rather sense the absence of self-MHC class I on tumor cells. HSP90 and HSP70 chaperones showed to be crucial for antigen presentation by MHC I molecule. Binder and colleagues reported that peptide antigens bound to HSPs, such as HSP90, HSP70, gp96, were presented 100 fold more efficiently by MHC I compared to free peptides (152, 153). Callahan and co-workers demonstrated that HSP90 inhibition disrupts the loading of peptides on MHC I (153). Furthermore, Kunizawa and Shastri showed that TCP-1 ring complex (TRiC/CCT) chaperonin is required for the expression of peptide-loaded MHC I on the cell surface (154). Later the same team found that inhibition of HSP90α or co-chaperone carboxyl terminus of Hsc70-interacting protein (CHIP) reduced presentation of peptide-bound MHC I on the cell surface (155).

Several investigators proposed the use of engineered NK cells as promising strategy for adoptive cell therapy in hematological malignancies (156). NK cells that are used in adoptive transfer can be allogeneic, autologous or immortalized such as NK-92 (156, 157). For research studies, NK cells can be isolated from peripheral blood or differentiated from stem cells, *ex vivo* expanded, activated with cytokines (IL-2, IL-15) and co-cultured with γ-irradiated feeder cells. It is important to point out that NK cells derived from peripheral blood mononuclear cells (PBMC) differ from NK cells differentiated from stem cells (156). For example, NK cells derived from cord blood showed no expression of CD57, high expression of NKG2A, lower expression of killer-immunoglobulin-like receptors (KIRs) and lower secretion of interferon-γ (156, 158). Several studies showed that NK cells can also be activated by HSP70 protein or 14-mer HSP70-derived peptide (TKD) in combination with IL-2 or IL-15 (**Figure 2**) (137–139). Multhoff and colleagues demonstrated that aggressive tumors have high expression of membrane-bound HSP70 (mHSP70) and that radio and/or- chemotherapy further increases surface expression of HSP70 (159–161). Furthermore, they reported that NK cells pre-activated with TKD and IL-2 recognize mHSP70 on tumor cells (137). Translating this to clinical trial, Multhoff et al. showed that four cycles of adoptive transfer of autologous NK cells pre-stimulated with TKD and IL-2 was well-tolerated and resulted in increase in the number of NK cells in peripheral blood of patients with mHSP70-positive non-small cell lung cancer (NSCLC) following radiochemotherapy in phase II clinical trial (140). Earlier, same research team demonstrated that NK cells activated with IL-2 and TKD and combined with anti-PD-1 antibody increased cytolytic activity of NK cells toward cancer cells and delayed tumor growth *in vivo* (162).

NK cells that express tumor-specific CARs showed to be efficiently applied in B cell malignancies (156). Currently, six CAR-NK therapies (CD19-CAR NK, CD22-CAR NK, CD19/CD22, CD7-CAR NK,CD19-t-haNK) are assessed in clinical trials for the treatment of lymphoma patients (156). Liu and colleagues used NK cells from the cord blood (CB) for incorporation of genes for CAR-CD19, IL-15 cytokine and caspase 9 as safety switch (iC9/CAR.19/IL15) to efficiently kill CD19-positive leukemia/lymphoma cells lines (156, 163). Same research group further assessed administration of HLA-mismatched iC9/CAR.19/IL15-transduced CB-NK cells in Phase I/II clinical trial to patients with r/r CD19- positive NHL and chronic lymphocytic leukemia (CLL) (164). Overall, 11 patients were administrated with anti-CD19-CAR NK cells, 8 patients (73%) achieved clinical response from which 4 patients with lymphoma and 3 patients with CLL had a complete remission (164). Notably, no neurotoxicity, CRS or increase in IL-6 were observed, which are frequently associated with the administration of CAR T therapies (156, 164).

### HSPs and Immune Checkpoints

Immunotherapy in the form of checkpoint modulation has advanced cancer research (165). In 2011, the first monoclonal antibody targeting immune checkpoint cytotoxic T-lymphocyte antigen-4 (CTLA-4) called ipilimumab received FDA approval. Later, monoclonal antibodies that target programmed death 1 (PD-1) such as pembrolizumab and nivolumab, and antibodies against PD-L1 such as atezolizumab and durvalumab were developed (165). Novel combinatorial approaches currently emerge, where the use of immune checkpoint modulators in combination with other anti-cancer therapies may further improve clinical response (166).

Taking into account that a major challenge in immunotherapy is a loss of tumor-associated antigens, HSP90 inhibitors were proposed as complementary approach to checkpoint inhibitors for cancer treatment (167). Rao and colleagues reported that inhibition of HSP90 resulted in proteasome-dependent degradation of its client oncoprotein EphA2 and, hence, increased tumor recognition by EphA2-specific CD8+T lymphocytes (168, 169). Haggerty and colleagues demonstrated that HSP90 inhibitors showed to upregulate the expression of tumor antigens such as Melan-A/MART-1, TPR-2, gp100 and enhance T cell recognition in melanoma cell lines (170). HSP90 client protein nucleophosmin-anaplastic lymphoma kinase (NPM/ALK) showed to induce PD-L1 *via* STAT3 activation in T cell lymphoma (171). Notably, administration of anti-PD-L1 antibody in combination with HSP90 inhibitor ganetespib showed significantly higher anti-tumor activity than when treated with anti-PD-L1 alone in syngeneic mouse models of melanoma and colon carcinoma (167). Furthermore, Mbofung and colleagues demonstrated that ganetespib in combination with anti-CTLA4 and anti-PD-1 antibodies improved survival and anti-tumor response in mice bearing MC38/gp100 tumors (172). Authors also showed that combinatorial treatment of HSP90 inhibitors and checkpoint blockade therapy decreased number of T regulatory cells and increased the expression of CXCL9, CXCL10 and IFN-γ (172). Combinatorial treatment also increased the number of CD8+T cells producing granzyme A and granzyme B, suggesting that combination of anti-CTLA4 and HSP90 inhibition enhances cytotoxic activity of CD8+T cells (172).

CD47 is an innate immune checkpoint highly expressed on the surface of tumor cells (93, 173). CD47 forms complex with signal regulatory protein α (SIRPα), which is expressed on phagocytic cells such as monocytes, macrophages and dendritic cells (93). CD47/SIRPα sends ‘don’t eat me” signals to innate immune cells, thus, inhibiting phagocytosis (174). Chao and colleagues reported that CD47 was overexpressed on samples derived from patients with various NHL subtypes (DLBCL,B-CLL, MCL, FL, MZL, pre-B ALL) and high CD47 expression correlated with poor clinical prognosis in NHL patients (173, 175). Blocking of CD47/SIRPα with anti-CD47 monoclonal antibody resulted in phagocytosis of acute myeloid leukemia cells (173). Combination of anti-CD47 monoclonal antibody (Hu5F9-G4) and rituximab showed promising results in patients with r/r DLBCL and FL in phase I clinical trial (176). Interestingly, Cook et al. demonstrated that inhibition of glucose-regulated protein-78 (GRP78), a member of HSP70 family, downregulated CD47 expression in tumor cells, leading to enhanced macrophage infiltration (177). Moreover, co-expression of CD47 and GRP78 showed to associate with poor survival in breast cancer patients (177). HSP90 also showed to play a role in CD47 regulation as inactivation of Myc, a client protein of HSP90, resulted in reduced expression of CD47 and PD-L1 (178, 179). It is interesting to note that HSP90 inhibitor PU-H71 induced apoptosis and inhibited tumor growth in patient-derived xenograft model of MCL *via* downregulating Myc (179, 180). Therefore, further studies are required to understand the HSP90-MYC-CD47/PD-L1 relationship and the role of GRP78 in CD47 regulation for the development of more specific and effective CD47-based therapies.

## Discussion

Lymphoma represents a unique group of cancer derived from major effector cells of an immune system such as B cells, T and NK cells (81, 93). Immunotherapy-based approaches showed encouraging results for patients with HL and NHL, however, severe toxicity and low efficacy profiles restrict the use of immunotherapy in lymphoma patients (2, 81). To overcome these limitations, various therapeutic strategies are developed that include the use of HSPs.

HSPs belong to evolutionally conserved family of chaperones that assist client proteins in folding, trafficking, degradation and showed to be involved in the most stages of cancer development (56, 181–184). High expression of HSPs on the surface correlates with the aggressiveness and resistance to therapy in many types of cancer (185, 186). Furthermore, HSPs are largely known for their critical role in regulating cell death mechanisms and immune responses (187–189).

Lymphoma cells create a complex and unique immune-modulatory tumor microenvironment, where inflammatory and stromal cells provide essential signals for growth, proliferation and survival of tumor cells (75, 95, 118). One of the major hallmark of lymphoma is represented by the deregulated critical signaling pathways including NF-kB, JAK-STAT, BCR signaling, PI3K/AKT, MAPK/ERK and apoptosis signaling pathways (82, 85–88, 190).Noticeably, specific members of HSP families, in particular, HSP90 showed to interfere with all these signaling cascades (83, 89, 103). Based on positive results from preclinical studies, several researchers proposed the use of HSP90 inhibitors for the treatment of lymphoma (122). However, result from phase II clinical trial of HSP90 inhibitor revealed low efficacy, but durable response, and acceptable toxicity profile in patients with r/r NHL (122). Taking into account critical role of HSP90 in lymphoma pathogenesis, further studies should be performed to assess the role of HSP90 in different subtypes of HL and NHL lymphomas.

For effective development of HSP-based therapy in lymphoma, it is critical to bear in mind that different HSP members reside in different cellular compartments where they perform specific functions (30, 191, 192). For example, mitochondrial HSP90 homolog known as tumor necrosis factor receptor-associated protein 1 (TRAP1) is involved in mitochondrial bioenergetics while endoplasmic reticulum (ER) HSP90 member referred to as glucose-regulated protein 94 (GRP94/gp96/Endoplasmin/HSP90B1) is critical for the unfolded protein response (193–196). It also appears that cell has some form of a balance of HSP distribution across compartments and cancer seems to impair this equilibrium, leading to the translocation of HSPs, which further reflects their functions (197–199). For example, surface expression of GRP94 showed to increase tumor immunogenicity and stabilize plasma membrane HER2 in breast cancer cells (200–202). So whether HSP-based immunotherapy also affects distribution of HSPs in HL and NHL, shifting HSPs from their primary locations is not yet clear and requires further investigation.

It is also important to point out that HSPs in extracellular milieu exist in several forms either secreted or membrane-bound and each form has distinct function (203–208). For example, dying tumor cells secrete HSP70s that serve as damage-associated molecular patters (DAMPs) and showed to elicit strong T cell response which with long-term exposure leads to the induction of immune tolerance and tumor growth (209–212). Conversely, viable tumor cells export HSP70 in exosomes which showed to activate myeloid-derived suppressor cells (MDSCs) and macrophages for the production of IL-6 and TNF-α, respectively (213, 214). Additionally, HSP70 on the surface of tumor cells serves as a recognition structure for NK cells (199, 215). In light of the reported, extracellular HSP70 activates T regulatory cells leading to the downregulation of interferon-γ and TNF-α section and upregulation of IL-10 and transforming growth factor-β (TGF-β) production (216). Along this line, interaction of extracellular HSP70 with antigen-presenting cells resulted in activation of NF-kB, leading to the production of TNF-α, IL-1β, IL-6 and IL-12 (56, 217–220).Figueiredo and colleagues reported that soluble HSP70 alone or in combination with IL-2 resulted in increased production of IFN-γ by T cells (221). Furthermore, stimulation of T cells with HSP70 and IL-2 or IL-7/IL-12/IL-15 resulted in upregulation of Granzyme B in CD4+T cells in target-independent manner, suggesting that extracellular HSP70 can induce target-independent cytotoxicity in T- helper cells (221). In light of the reported, extracellular HSP110 induce pro-inflammatory phenotype in macrophages (222). Along this line, HSP27-positive tumor –derived exosomes enhance immunosuppressive activity of MDSCs whereas soluble HSP27 induces tolerogenic phenotype in macrophages (223, 224). Furthermore, extracellular HSP27 inhibits differentiation of monocytes to DCs (225). Therefore, taking into account immunologic role of extracellular HSPs, it is important to study their functions in lymphoma pathogenesis and further monitor expression of HSPs in different stages and subtypes of HL and NHL.

Despite their extracellular roles in tumor immunology, HSP members have also distinct intracellular immunologic functions. For example, ER HSP90 homolog GRP94 plays important role in immunosuppressive activity of T regulatory cells, in lymphopoiesis of T and B cells, in production of proinflammatory cytokines by tumor-associated macrophages, in the regulation of platelet GPIbα subunit of GPIb-IX-V complex and maturation of dendritic cells (195, 201, 202, 226–231). Along this line, mitochondrial HSP70 homolog GRP75/mtHSP70/mortalin/HSPA9 showed to interact with complement C9 and protect tumor cell from complement-dependent cytotoxicity (232–234).Furthermore, HSPs regulate an important component of innate immune response- the Nod-like receptor protein-3 (NLRP3) inflammasome (235, 236). Therefore, taking into account that central role in lymphoma pathogenesis play immune evasion mechanisms, intracellular immunologic roles of HSPs should also be considered for the development of more effective and safe HSP-based immunotherapy for the HL and NHL treatment.

HSP chaperones also showed to interact with each other. For example, mortalin interacts with HSP60 and HSP90 whereas GRP94 interacts with binding immunoglobulin protein (BiP/GRP78/HSPA5) (234, 237–239). Furthermore, individual homologs may have specific client networks. For example, ER HSP90 member GRP94 has client network that does not overlap with client network of cytosolic HSP90 homologs (195, 240, 241). Conversely, members from different HSP families may have overlapping client network. For example, HSP110 and HSP90 showed to stabilize c-Myc and Bcl-6 (109, 120, 179). Therefore, it is critical to note that blocking HSP member may further affect its co-chaperones, its client network and other HSP chaperones. Therefore, further studies should explore what happens on the level of individual HSP members in different subtypes of HL and NHL lymphoma and whether specific blocking of a particular homolog and, hence, its client and co-chaperone network, will be more advantageous for the lymphoma treatment.

HSP90 showed to affect CD3 and CD28, thus, the effect of HSP90 on CAR- containing CD3 and CD28-derived domains requires further investigation (133). From the other hand, HSP70-derived peptide TKD has been used for *ex vivo* activation of NK cells for adoptive transfer therapy and, since no severe toxicity was observed for NK-based immunotherapy, this strategy may be exploited for the development of lymphoma immunotherapy (140). In light of the reported, two research teams proposed to target membrane-bound HSP70 and HSP90 isoforms on tumors by CARs (134, 136).

Evidently, HSP90 *via* its client network (NPM/ALK and Myc) showed to be involved in the regulation of immune checkpoints such as PD-L1 and CD47 whereas HSP70 ER member GRP78 showed to be co-expressed with CD47 (171, 177–179). Since combination of HSP90 inhibitors with either anti-PD-1, anti-PD-L1 or anti-CTLA-4 antibodies showed anti-tumor effect in mouse models, combinatorial approaches of using HSP90 inhibitors and checkpoint inhibitors or HSP70 inhibitors coupled with anti-CD47 antibodies may further improve anti-tumor response (167, 172).

Another strategy to improve therapy responses involves the use of biomarkers that can predict clinical outcome. Large body of evidence suggests that extracellular HSPs can be used as predictive, prognostic and diagnostic biomarkers of cancer (31, 63, 64, 242–244). Further studies should be performed to assess expression of HSPs in extracellular milieu in their potential to predict clinical response in patients with HL and NHL. Recently, Dunphy and colleagues have conducted phase I clinical trial to test the safety and feasibility of administering ^124^I-PU-H71 radiologic agent followed by positron emission tomography (PET) to detect HSP90 within epichaperome complex in various types of tumors, thus supporting further development of HSP90-based targeted- therapeutics (245).

Taken together, members of HSP family may be exploited for the development of more efficacious treatment, though, further studies are required to understand the effect of HSP expression in various lymphoma subtypes and their use in the development of T/NK-based immunotherapies and combination approaches. Moreover, the role of HSPs as biomarker to predict clinical outcome in lymphoma patients warrants further investigation.

## Conclusion

Lymphoma is a heterogeneous group of cancer, derived from immune cells and characterized into two major subtypes such as Hodgkin and Non-Hodgkin lymphoma. HSP members in particular HSP90, HSP60 and HSP70 are highly expressed in most subtypes of HL and NHL lymphoma. Evidently, HSPs play a major role in hallmarks of lymphoma pathogenesis including their involvement in immune evasion and dysregulation of key signaling cascades. Exploiting HSPs in immunotherapy-based approaches and as biomarkers for the lymphoma therapy may prove effective, however, requires further investigation.

## Author Contributions

ZA: conceptualization and manuscript writing. YM: manuscript editing. AS: Resources. All authors contributed to the article and approved the submitted version.

## Funding

This work was funded by RFBR, project number 20-315-90081. YM was supported by the RAKFOND grant 2020-02.

## Conflict of Interest

The authors declare that the research was conducted in the absence of any commercial or financial relationships that could be construed as a potential conflict of interest.
